# Water Flux Prediction in Direct Contact Membrane Distillation Subject to Inorganic Fouling

**DOI:** 10.3390/membranes12020157

**Published:** 2022-01-28

**Authors:** Francisco Suárez, María B. del Río, Jazmín E. Aravena

**Affiliations:** 1 Departamento de Ingeniería Hidráulica y Ambiental, Pontificia Universidad Católica de Chile, Santiago 7820436, Chile; mbelendelrio@gmail.com; 2 Centro de Excelencia en Geotermia de Los Andes (CEGA), Santiago 7820436, Chile; 3 Centro de Desarrollo Urbano Sustentable (CEDEUS), Santiago 7820436, Chile; 4 Independent Researcher, Santiago 7820436, Chile; jearaven@gmail.com

**Keywords:** direct contact membrane distillation, fouling, cake filtration model, distillate flux decline, heat and mass transfer modeling, scanning electron microscopy, energy-dispersive X-ray spectroscopy

## Abstract

Freshwater is a limited resource, which has driven the development of new purification and water-reuse technologies. One promising technology for water treatment is membrane distillation (MD). One of the main problems of MD, and of many desalination technologies, is membrane fouling, which reduces the performance of the membrane. This work presents a mathematical model that aims to predict distillate fluxes in direct-contact MD when fouling occurs as salts are deposited onto the membrane surface, forming an inorganic fouling layer. The mathematical model uses a heat- and mass-transfer formulation for prediction of the distillate flux under steady state conditions, and it is combined with the cake-filtration theory to represent the distillate fluxes after the onset of membrane fouling. Model results agree well with experimental observation of distillate fluxes, both before (~12–14 kg m^−2^ h^−1^) and after the onset of membrane fouling, with root-mean-square errors smaller than 1.4 kg m^−2^ h^−1^ in all the experiments. These results suggest that the cake-filtration theory can be used to represent water flux decline in MD membranes prone to inorganic fouling. From our experiments and from the modelling exercise, we found that the onset of membrane failure was relatively constant; the precipitation reaction constant is conditioned by the physicochemical interaction between the feed solution and the membrane; and the rate of flux decline after membrane fouling depends on flow conditions as well as on the precipitation compound. However, the proposed model has limitations that must be addressed in future investigations to validate it under a wider range of operating conditions, for membranes composed by other materials and with different feed solutions to address organic, biological, and/or colloidal fouling, which typically occur under real conditions.

## 1. Introduction

The increase in population and living standards results in a greater demand for water consumption. Since the 1960s, water scarcity has grown to a great extent, and the proportion of the world’s population living under chronic water scarcity (<1000 m^3^/per capita/year) rose from 9% (280 million people) in 1960 to 35% (2.3 billion) in 2005 [1]. Climate change also exacerbates this problem by changing precipitation and evaporation patterns, which in turn modify freshwater resources collected from rivers and aquifers [2,3,4,5].

**Figure 1:**
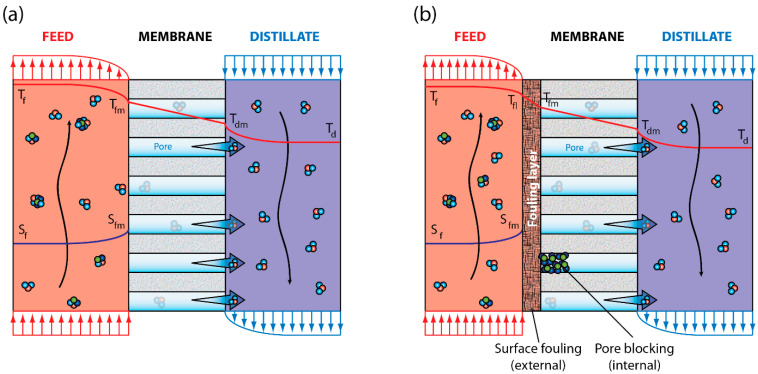
Temperature (*T*) and concentration (*S*) profiles in a direct contact membrane distillation module: (**a**) system operating without fouling and (**b**) system that exhibits a fouling layer (surface fouling) and pore blocking. Subindices *f*, *d*, and *m* correspond to feed, distillate, and membrane, respectively, whereas *fl* is the fouling layer.

Desalination technologies are considered the main solutions to water scarcity [6,7,8,9,10,11,12], which become more attractive when they are driven by renewable energy sources [9,13,14,15,16]. On one hand, the most used desalination technologies use reverse osmosis (RO), multi-effect distillation (MED), and multi-stage flash (MSF) [17,18,19,20]. On the other hand, most of the emerging technologies use forward osmosis (FO) and membrane distillation (MD) [13,21,22]. MD is a desalination technique that works at low temperatures, i.e., with low energy requirements [13,23,24,25,26]. Therefore, its potential to be coupled with waste heat or solar energy is large [13,26,27,28]. The MD module can have different configurations [13], with the direct contact membrane distillation (DCMD) configuration being the most common, as it is simple to operate and it has low investment costs compared to other membrane separation technologies when driven by renewable energy [13,14,15,29,30,31,32]. In DCMD, the water (and other volatile compounds) of the feed solution evaporates at the membrane surface and crosses a hydrophobic membrane as water vapor (Figure 1a). Then, water vapor condensates at the distillate side of the membrane module, producing a highly pure distillate [13,33].

One of the main issues in membrane-based processes is fouling [7,24,34,35,36]. Membrane fouling refers to the accumulation of unwanted deposits at the membrane surface or within the pores of the membrane (Figure 1b), which reduces the flow of distilled water and the performance of salt rejection. Some of the negative effects of fouling include membrane wetting, salt layer adsorption, pore blocking, and cake formation [13,37,38]. When MD membranes are not wet, they act as a barrier to non-volatile solutes dissolved in the feed solution. However, when the MD membranes are partially or fully wet, the feed solution flows through the pores of the membrane, leading to a poor-quality distillate solution [7,13,39,40,41]. According to Tang et al. [42], the factors that contribute to membrane fouling are related to the characteristics of the feed solution and precipitate, membrane properties, and operating conditions. Therefore, to improve MD performance, it is important to understand how these factors interact [13].

Several efforts have been made to address the fouling problem, especially focusing on how to clean the membranes [7,24,29,36]. However, effective fouling control techniques for MD have not yet been developed [40]. Current techniques for fouling control are feed pretreatment and membrane cleaning [25,39,43,44,45]. Some other approaches to mitigating fouling include fabricating new membranes with new designs and materials [41]; changing flow regimes; and developing antifouling membranes, including modifying the membrane surface, designing of new membrane modules, and the use of physical and chemical techniques [7,13,45,46,47].

Most of the published research associated with modeling of MD systems investigates transport phenomena in boundary layers and through the membrane, while very little attention has been paid to fouling phenomena [13,24,37,47]. However, in other filtration systems, attempts have been made to model fouling. For example, the cake filtration model assumes that fouling begins after an initial deposit of the precipitate sticks on the membrane surface [48,49]. The cake filtration theory considers that the fouled region of the membrane is partially permeable to the water flow. As more unwanted deposits accumulate at the membrane surface, the salts begin to deposit directly on the fouling layer, causing an increase in hydraulic resistance to flow associated with the formation of the cake layer [48].

Due to the scarcity of studies that attempt to model the fouling phenomenon in MD, this work aims to develop a simple methodology based on the cake-filtration theory to represent water flux decline in a DCMD membrane subject to inorganic fouling. The specific objectives of this work are to: (i) present the mathematical model that represents the effect of inorganic fouling on the performance of an MD system; (ii) validate the mathematical model with laboratory experiments; and (iii) present a qualitative analysis of the precipitate that sticks to the membrane surface. The scope of this work is limited to inorganic fouling, also known as scaling or precipitation fouling, without considering membrane wetting.

## 2. Materials and Methods

### 2.1. Experimental Setup

**Figure 2:**
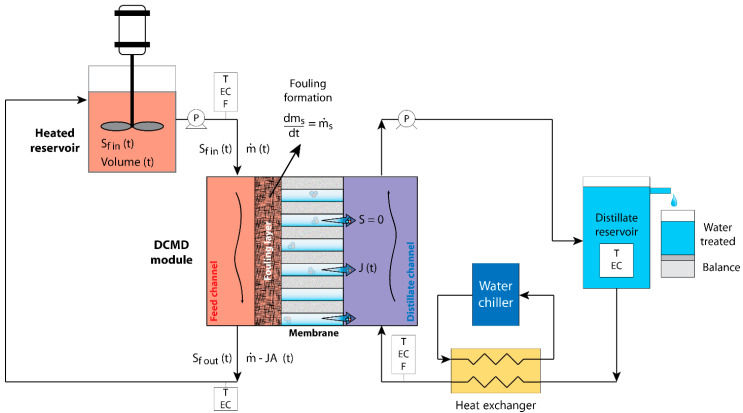
Diagram of the experimental setup showing the heated reservoir and the direct contact membrane distillation module, as well as the distillate stream recirculation. Concentrations and fluxes, as a function of time (t), are also shown. Note that *J* is distillate flux and *A* is the membrane area. The symbols P, EC, F, and T represent pumps, electrical conductivity probes, flow meters, and temperature sensors, respectively.

The experimental setup, which is depicted in Figure 2, corresponds to a typical batch reactor used in desalination experiments [33]. It consists of a heated reservoir, i.e., a constant temperature tank, with an initial volume of feed solution, which can be modeled as a completely stirred-tank reactor (CSTR). The feed solution is pumped towards the MD module, passes through the feed channel, where it is concentrated as a fraction of it passes through the membrane, and then is poured again into the heated reservoir. As a consequence of this process, the feed solution volume decreases in the reservoir, whereas its concentration increases. Temperature and electrical conductivity were measured in different locations within the experimental setup (Figure 2) using the 4320-bench conductivity meter (Jenway Ltd., Essex, UK). Pressure within the DCMD system was not measured as we used the same configuration as Cath et al. [33], in which the system operated near atmospheric pressure (approximately 0.94 atm). Note that Figure 2 presents the different variables involved in the water and salt mass conservation principle that are used in the next section to describe the heat and mass transfer model that accounts for fouling.

### 2.2. Heat and Mass Transfer Model for DCMD with Fouling

The water flux across the membrane for a given time, J(t) (kg m^−2^ s^−1^), can be expressed as:

(1) $$J\left(t\right)=\{\begin{array}{ll} J_{0} & if\;\;t\leq t_{c}\\ \alpha \left(t\right)\;J_{0} & if\;\;t>t_{c} \end{array}\;$$

where $J_{o}$ (kg m^−2^ s^−1^) is the water flux through the clean membrane; $t_{c}$ (s) is a critical time, defined here as the time of the onset of membrane failure; and α(t) (-) is a time-dependent flux reduction parameter taken from the cake filtration theory [37,49]:

(2) $$\alpha \left(t\right)={\left(1+k_{f}\left(t-t_{c}\right)\right)}^{-1/2}\;$$

where $k_{f}$ (s^−1^) is a coefficient that depends on the flow conditions and the solution properties, and it is typically obtained by calibration; i.e., is a fitting parameter that allows reproducing the observed data using a model [37,50]. Below, we describe how to determine $J_{o}$, as well as how to constrain the water flux reduction with the precipitated salts at the feed side of the membrane surface. Note that this formulation is analogous to definitions of time-dependent heat and mass transfer resistances [37].

To estimate $J_{o}$, i.e., the water flux before fouling occurs or after the membrane is cleaned, the steady state heat and mass transfer model developed by Suárez et al. [14] is used. This model assumes that the pores of the membrane surface are at liquid–vapor equilibrium, and estimates $J_{o}$ as:

(3) $$J_{o}=C_{m}\left[p^{0}\left(T_{fm}\right)\left(1-\chi \left(S_{fm}\right)\right)\xi \left(T_{fm},\;S_{fm}\right)-p^{0}\left(T_{dm}\right)\right]$$

where T (°C) is temperature, S (%*w*/*w*) is the solute concentration, $C_{m}$ (kg m^−2^ s^−1^ Pa^−1^) is the membrane distillation coefficient, $p^{0}\left(T\right)$ (Pa) is the vapor pressure of the pure substance, χ(S) (-) is the mole fraction of the solute, and ξ(T ,S) (-) is the activity coefficient [23]. The subindices *fm* and *dm* represent the feed and distillate sides of the membrane surface, respectively. Because the vapor transport across the membrane pores generally occurs through combined molecular and Knudsen diffusion processes, the $C_{m}$ can be represented by [14,51]:

(4) $$C_{m}=\frac{\phi }{\tau \delta }\;\frac{M}{RT}{\left[\frac{p_{a}}{PD_{wa}}+\frac{1}{D_{k}}\right]}^{-1}$$

where ϕ (-) is the membrane porosity, τ (-) is the membrane tortuosity, δ (m) is the membrane thickness, M (kg mol^−1^) is the molecular weight of water, R (J °C^−1^ mol^−1^) is the gas constant, $p_{a}$ (Pa) is the partial pressure of air entrapped in the pores, P (Pa) is the total pressure inside the pores, $D_{wa}$ (m^−2^ s^−1^) is the diffusion coefficient of water vapor in air, and $D_{k}$ (m^2^ s^−1^) is the Knudsen diffusion coefficient. To estimate *J* through the membrane, $S_{fm}$, $T_{fm}$, and $T_{dm}$ must be determined.

$S_{fm}$ can be estimated from a mass balance in the concentration boundary layer of the feed side channel [52]:

(5) $$S_{fm}=S_{f}exp\left(\frac{J}{\rho_{f}\;K}\right)\;$$

where $\rho_{f}$ (kg m^−3^) is the feed solution density, K (m s^−1^) is the film mass transfer coefficient (see Appendix A for a description of the mathematical formulation used to determine K), and $S_{f}$ (%*w*/*w*) is the solute concentration in the bulk feed. The previous equation assumes a 100% solute rejection by the membrane.

$T_{fm}$ and $T_{dm}$ can be estimated using a steady state heat transfer analysis, where the convective heat transfer in the feed ($q_{f}$, W m^−2^) and distillate ($q_{d}$, W m^−2^) boundary layers and the heat transferred across the membrane ($q_{m}$, W m^−2^) are equal [14]:

(6) $$q_{f}=h_{f}\left(T_{f}-T_{fm}\right),$$

(7) $$q_{d}=h_{d}\left(T_{dm}-T_{d}\right),$$

(8) $$q_{m}=\left(\frac{k_{m}}{\delta }+\frac{JH_{v}\left(T\right)}{\Delta T_{m}}\right)\Delta T_{m}=\left(h_{c}+h_{v}\right)\Delta T_{m},$$

where $h_{f}$ and $h_{d}$ (W m^−2^ °C^−1^) are the heat transfer coefficients in the feed and distillate sides of the membrane, respectively (see Appendix A for a description of the mathematical formulation used to determine $h_{f}$ and $h_{d}$); $T_{f}$ and $T_{d}$ (°C) are the bulk temperatures in the feed and distillate channels, respectively; $k_{m}$ (W m^−1^ °C^−1^) is the effective thermal conductivity of the membrane; $H_{v}\left(T\right)$ (J kg^−1^) is the latent heat of vaporization; $\Delta T_{m}=T_{fm}-T_{dm}$ (°C); and $h_{c}$ and $h_{v}$ (W m^−2^ °C^−1^) are the heat transfer coefficients for conduction and vapor flow across the membrane, respectively. The mass and heat transfer coefficients previously defined can be determined using empirical correlations for different flow regimes. In this work, we used the parametrizations described in [50]. $T_{fm}$ and $T_{dm}$ can be found using the following equations [14]:

(9) $$T_{fm}=T_{f}-\frac{\left(T_{f}-T_{d}\right)h_{f}{}^{-1}}{h_{f}{}^{-1}+h_{d}{}^{-1}+{\left(h_{c}+h_{v}\right)}^{-1}},$$

(10) $$T_{dm}=T_{d}+\frac{\left(T_{f}-T_{d}\right)h_{p}{}^{-1}}{h_{f}{}^{-1}+h_{d}{}^{-1}+{\left(h_{c}+h_{v}\right)}^{-1}},$$

When estimating the water flux reduction across the membrane, it is important to constrain this flux with the physicochemical processes that occur at the membrane module. Therefore, the water fluxes across the membrane must also be coherent with the mass of precipitated salts at the membrane feed-side surface. Salt precipitation was modeled using the following reaction kinetic [53]:

(11) $$\frac{dm_{s}}{dt}={\dot{m}}_{s}=k_{pr}{\left(S_{f}-S_{c}\right)}^{n},$$

where $m_{s}$ (kg) is the mass of precipitated salts at the membrane; t (s) is the time; ${\dot{m}}_{s}$ (kg s^−1^) is the mass rate of precipitated salts; $k_{pr}$ (kg s^−1^) is the precipitation reaction constant; $S_{c}$ (%*w*/*w*) is the feed solution concentration at $t_{c}$; and n (-) is the reaction order. The salt mass balance in the feed channel of the membrane module, for a defined time step, can be expressed as [50]:

(12) $$S_{f\;in}\;\dot{m}-S_{f\;out}\left(\dot{m}-JA\right)=k_{pr}{\left(S_{f}-S_{c}\right)}^{n},$$

where $S_{f\;in}$ and $S_{f\;out}$ (%*w*/*w*) are the inlet and outlet concentrations in the feed channel; $\dot{m}$ (kg s^−1^) is the mass flow rate at the inlet of the feed channel; and A (m^2^) is the membrane area. In our experiments, we tested n = 0.5, 1, 2, and 3 and we calibrated $k_{pr}$ so that the mass balance of precipitated salts was fulfilled. n=0.5 yielded the best result to satisfy the mass balance of precipitated salts throughout all our experiments [50].

The previous equations are solved in an iterative manner and in conjunction with the water and salt balance in the reactor. To begin the computations, the module dimensions (e.g., hydraulic diameter of channels, membrane area) and the operating conditions (e.g., $\dot{m}$, initial solute concentration, and solution volume in the reactor) are needed.

### 2.3. Membrane Module and Laboratory Experiments

#### 2.3.1. Membrane Module

The membrane module used in this investigation has symmetric channels on both sides of the membrane. Each flow channel is 200 mm long, 50 mm wide, and 3 mm high. We utilized a TS22 polytetrafluoroethylene (PTFE) membrane with pore size of 0.22 mm, porosity of 70%, thickness of 175 mm, and area of 139 cm^2^ [33].

#### 2.3.2. Laboratory Experiments

The objective of the experiments was to validate the proposed model that predicts water flux decline in DCMD when fouling occurs. Thus, we performed experiments in which the membrane was fouled and then cleaned. At the beginning of each experiment, a new membrane was used. When the water flux approached zero, the distillation process was halted, and the membrane was cleansed with a determined cleaning solution for 30 min and then rinsed with 12 L of double-distilled water. The additional cleaning using double-distilled water was performed to ensure that no cleaning solution was in the feed channel of the membrane module before the next fouling test. Table 1 presents the details of each experiment and its corresponding cleaning procedures. The synthetic feed solutions that were used in the experiments aimed to mimic calcium sulfate (CaSO_4_) deposition onto the membrane surface, as CaSO_4_ typically results in membrane fouling [7,12,39,41]. As shown in Table 1, each experiment consisted in a series of trials in which the same cleaning solution was used, but the cleaning solutions between experiments 1 and 2 differ.

The operating conditions were similar for all the experiments. The temperatures of the feed and distillate solutions were ~40 and ~20 °C, respectively (Table 1). These temperatures were reached after some time from the beginning of the experiment. The volumetric flow rates of the feed and distillate solutions were 1.5 L/min.

### 2.4. Microscopy

With the aim of performing a qualitative analysis, the membranes utilized in the experiments were observed with a scanning electron microscope (SEM). Additionally, energy-dispersive X-ray spectroscopy (EDS) was performed to obtain concentration of the elements that make up the sample. For this, the CaSO_4_ feed solution of experiment 2 (Table 1) was employed, and samples were collected for the following situations: (1) after distilling the feed solution prior to membrane cleaning and (2) after the membrane was washed five times.

## 3. Results

### 3.1. Water Flux Prediction

#### 3.1.1. Experiment 1

**Figure 3:**
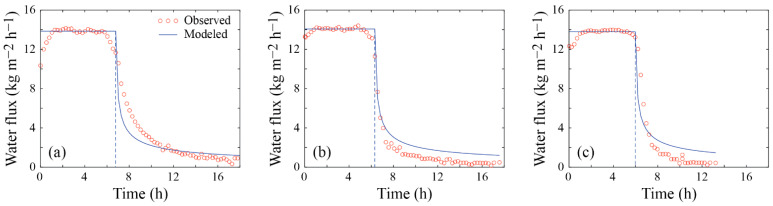
Observed and modeled water fluxes across the membrane in Experiment 1: (**a**) system’s performance with a brand-new membrane; (**b**) system’s performance after first cleaning; (**c**) system’s performance after second cleaning.

The water flux across the membrane for Experiment 1 is presented in Figure 3. Steady-state conditions are observed for up to 6–7 h of operation, regardless of whether the membrane was brand new (Figure 3a) or was cleaned (Figure 3b,c). Water fluxes ranging between 13.7 and 14.1 kg m^−2^ h^−1^ were observed during steady state conditions. Therefore, changes of less than 2% in the magnitude of the water flux across the membrane occurred among cleaning cycles and are explained by small temperature variations of ~0.1–0.2 °C in the bulk feed and distillate streams. Under these conditions, the temperature polarization coefficient, $TPC=T_{fm}-T_{dm}/T_{f}-T_{d}$, and the concentration polarization coefficient, $CPC=S_{fm}/S_{f}$), are ~0.86 and ~1.02, respectively. These values suggest that the system operates near efficient conditions [33].

Model predictions agree fairly well with the observed data for the steady-state conditions, i.e., up to the critical times. Following the critical time, flux reduction was more drastic after the cleaning cycles compared to the first experiment performed with the new membrane. When the membrane was brand new, the modeled fluxes decreased more rapidly than the experimental fluxes, whereas the opposite was observed after the first cleaning cycle. In general, the modeled flux reduction agrees well with the observed data—with a root-mean-square error (RMSE) of the water flux in the entire experiment of ~1.4 kg m^−2^ h^−1^. These results are obtained with $k_{f}$ = 3.31 × 10^−3^ s^−1^ in each subset of experiments, as the membrane, the water flow in the membrane channels, and the feed solution are the same. In this subset of experiments, as depicted in Table 2, the precipitation reaction constants, $k_{pr}$, are 1.12 × 10^−3^, 9.34 × 10^−4^, and 1.39 × 10^−3^ kg h^−1^, respectively. Using the previous values of $k_{pr}$, the corresponding errors between experimental and modeled salt precipitation are 4.95 × 10^−10^, 1.45 × 10^−11^, and 1.55 × 10^−10^ kg, which are much smaller than the initial mass of salts incorporated in the feed solution (4.55 × 10^−2^ kg). As shown in Table 2, the recovery rate (R) for this subset of experiments ranged between 34% and 42% (Table 2), and the electrical conductivity of the distillate solution remained at <1 µS cm^−1^, suggesting that membrane wetting did not occur. Note that the experimental data obtained from this experiment are available in the Supplementary Material.

#### 3.1.2. Experiment 2

**Figure 4:**
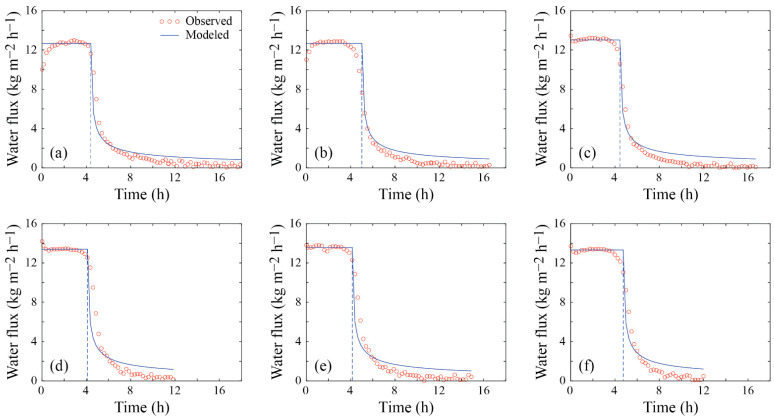
Observed and modeled water fluxes across the membrane in Experiment 2: (**a**) system’s performance with a brand-new membrane; (**b**) system’s performance after first cleaning; (**c**) system’s performance after second cleaning; (**d**) system’s performance after third cleaning; (**e**) system’s performance after fourth cleaning; (**f**) system’s performance after fifth cleaning.

The results of Experiment 2 are presented in Figure 4 and Table 2. For this subset of experiments, as shown in Figure 4, steady-state conditions are observed up to ~4–5 h of operation, with water fluxes higher than ~12 kg m^−2^ h^−1^. Similarly to Experiment 1, water fluxes before membrane fouling are very similar, regardless of whether the membrane was brand new (Figure 4a) or had been cleaned (Figure 4b–f), and *TPC* and *CPC* were ~0.87 and 1.02, respectively. Slight differences between the water fluxes between experiments 1 and 2 are explained by small temperature variations of ~0.1–0.2 °C in the bulk feed and distillate streams, as well as by the different composition of the feed solution.

In this experiment, model predictions also agree fairly well with the observed data up to the critical times (Figure 4). After the onset of membrane fouling, the model predicts well the water flux reduction, although it slightly overestimates the final fluxes observed in the experiments (RMSE of ~1.3 kg m^−2^ h^−1^ for the entire experiment). Nonetheless, we found that $k_{f}$ was 4.88 × 10^−3^ s^−1^, which is very similar to the $k_{f}$ value obtained in Experiment 1. This result suggests that $k_{f}$ most likely depends on the flow conditions, the precipitated compound (CaSO_4_), and the membrane characteristics, which were the same in both experiments. As shown in Table 2, the errors in the precipitated mass of salts on the membrane are on the order of 10^−10^ kg, which is negligible when compared to the initial mass of salt added to the feed solution (3.25 × 10^−2^ kg). In these experimental tests, the R was ~25% (Table 2), and similarly to Experiment 1, the electrical conductivity of the distillate solution remained at <1 µS cm^−1^, i.e., membrane wetting was not observed (the experimental data are presented in the Supplementary Material).

### 3.2. Microscopy

**Figure 5:**
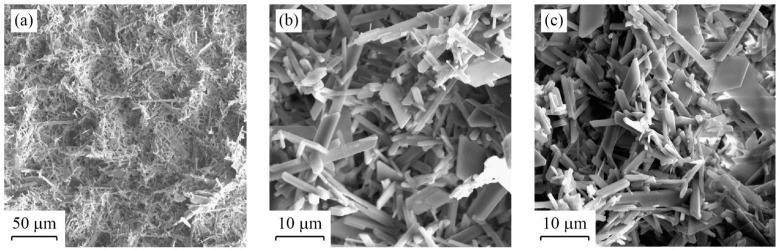
SEM images of the fouled membrane after distilling the feed solution (Experiment 2 without cleaning): (**a**) 400× zoom (50 μm), which shows that the CaSO_4_ crystals are deposited throughout the membrane surface; (**b**,**c**) 2000× zoom (10 μm) at two different locations of the membrane.

**Figure 6:**
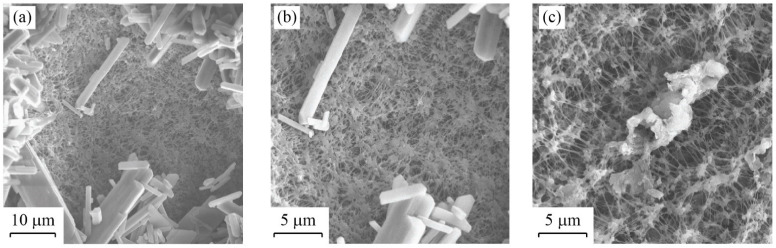
SEM images of the membrane after the fifth cleaning cycle (end of Experiment 2): (**a**) 2000× zoom (10 μm) where both CaSO_4_ crystals and the membrane surface are observed; (**b**) 4000× zoom (5 μm); (**c**) 4000× zoom (5 μm) in which a sulfur deposit over the membrane is observed.

The SEM images obtained on a membrane after desalting the feed solutions are shown in Figure 5 and Figure 6. The images presented in Figure 5 were taken after the membrane was fouled, without exposing the membrane to a cleaning cycle. Figure 5a depicts a 400×-zoom image, which reveals that the CaSO_4_ crystals precipitate throughout the membrane surface. Some locations in the membrane have a thicker CaSO_4_ crystal layer than other locations, although in general the precipitates distribute evenly. Figure 5b,c correspond to a 2000×-zoom image that clearly depict the CaSO_4_ crystals that precipitate over the membrane. Figure 6 presents SEM images of the membrane after the fifth cleaning cycle (i.e., at the end of Experiment 2). Both CaSO_4_ crystals and the membrane surface are observed in Figure 6a,b, whereas a sulfur (S) deposit, which is larger than the membrane pores, is embedded in the membrane, as shown in Figure 6c. These images suggest that, even when the membrane is not 100% cleaned after a cleaning cycle, it can achieve a similar performance in terms of the water flux magnitude compared to the brand-new membrane.

**Figure 7:**
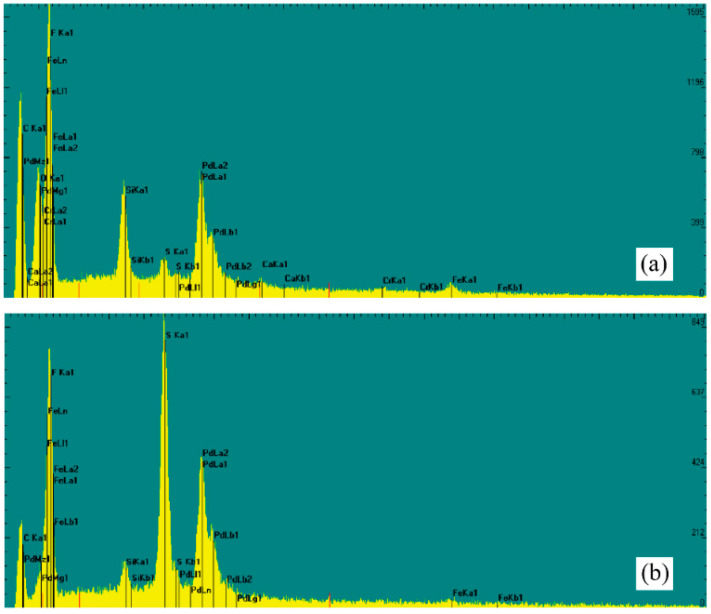
SEM-EDS analysis: (**a**) membrane after the fifth cleaning cycle, (**b**) membrane after the fifth cleaning cycle with the sulfur deposit.

Figure 7 shows the results of the SEM-EDS analysis of the membrane deposits in two different locations after the fifth cleaning in Experiment 2. The fluorine (F) and carbon (C) concentrations are attributed to the membrane composition, which is made of PTFE. When comparing Figure 7a,b, a large variability in sulfur (S) is observed. The SEM-EDS analysis presented in Figure 7b corresponds to the S deposit presented in Figure 6c.

## 4. Discussion

With the aim of investigating technological solutions that mitigate water scarcity, this work presents a simple mathematical formulation to predict water flux decline in DCMD subject to fouling. The formulation is based on a steady state heat and mass transfer model, combined with the cake-filtration theory to represent the transient conditions observed during fouling. Water flux prediction before membrane fouling occurred, i.e., under steady-state conditions, was very good. This performance was expected as these types of models have shown successful results in a wide range of applications [14,15,23,27,31,37]. Water flux prediction after the onset of membrane fouling was also acceptable, although the model slightly overestimates the observed water flux (e.g., see Figure 3 and Figure 4).

Our results suggest that the cake-filtration theory can be used to represent water flux decline in membranes prone to fouling. Nonetheless, the proposed model uses three parameters that must be calibrated to obtain successful results. First, the critical time (*t_c_*) is an important parameter in the proposed model as it defines the onset of fouling. In the CaSO_4_ experiments, *t_c_* was relatively constant, and no clear trend is observed after more cleaning cycles were carried out. This is an important parameter that should be further investigated with more fouling studies to help to parametrize it better. Second, the model also requires the knowledge of the precipitation reaction constant ($k_{pr}$). $k_{pr}$ represents the precipitation rate of the mass of salts in the membrane, which is conditioned by the physicochemical interaction between the solution and the membrane. As shown in Table 2, the error in the precipitated salts from our experiments suggests that $k_{pr}$ can be obtained with confidence by performing a mass balance considering the corresponding reaction kinetic—see Equations (11) and (12). This parameter is important as it allows one to successfully estimate the failure of the system due to the accumulation of salts and its effect on the performance of the membrane. Hence, it deserves to be further explored in future works. $k_{f}$ is the third parameter that must be calibrated to obtain acceptable water flux predictions under fouling conditions. Even though in our experiments we can only claim that $k_{f}$ is an empirical parameter, our results suggest that it is likely that it depends on the flow conditions and the precipitated compound, i.e., CaSO_4_, for our experiments. This result agrees with those reported by Srisurichan et al. [37]. Therefore, future investigations to improve the work presented in this manuscript should aim to validate the mathematical model under a wider range of operating conditions using different feed solutions that will form other precipitates and for membranes composed of other materials. In this way, the information available to expand the scope of this model will include all that described by Tang et al. [42], which should cover the main factors that contribute to membrane fouling.

Another limitation of our modeling approach is that it does not consider membrane wetting; crystal growth in the membrane pores; or organic, biological, or colloidal fouling [7,13,54]. In our experiments, we did not observe a detriment in the quality of the distilled solution, and thus, our results suggest that membrane wetting did not occur and that salt crystals were likely formed at the membrane surface and were removed by the cleaning process. Additionally, our results also suggest that for the conditions tested, the cleaning process did not have an impact on the membrane properties. Even though the investigation of the impact of the cleaning procedure on membrane properties and distillate fluxes is out of the scope of this work, we hypothesize that the cleaning process used in this research was excellent and allowed us to mostly recover the brand-new membrane fluxes because we used synthetic feed solutions that only formed scaling. Other unpublished experiments that we have performed with real raw water have not been as successful as the results presented in this work. The lower cleaning efficiency obtained when using real raw water is extensively documented in the scientific literature [39,40,41]. Therefore, it is important to recall the limitations of this work, as our model assumes inorganic fouling at the membrane surface, and our observations suggest that this was the case in the experiments that were performed. Nonetheless, this may not be the case when working with real conditions, in which organic, biological, and colloidal fouling can also occur [54]. These foulants can not only reduce the permeability through the membrane but also decrease its hydrophobicity. Moreover, fouling also reduces the lifecycle efficiency of the membrane, which in turn results in an increase in the energy usage, an increase in the frequency of membrane cleaning and/or membrane substitution, and consequently an increase of the maintenance costs of the overall process [54]. Note also that the cleaning agents must be selected carefully, as they also may influence membrane properties [47,54,55]. The impact of other types of fouling, as well as of the cleaning agent, on membrane properties must be explored in future investigations.

The SEM analysis presented in Figure 5 suggests that CaSO_4_ crystals were distributed evenly throughout the membrane surface, although with different thicknesses of the fouling layer. This result strengthen our approach of using the cake-filtration theory to model the fouling layer [37,48,49]. Nonetheless, we acknowledge that at the onset of membrane fouling there must be large variability during the crystal formation process, both in the bulk feed supersaturated solution and when surface nucleation occurs [56]. Therefore, as opposed to the results obtained in our experiments, we expect that, for different types of precipitates and operating conditions, the critical time (*t_c_*) may also have large variability. The SEM images presented in Figure 6 also highlight that, even when the membrane is cleaned and rinsed, there are locations at which deposits still adhered to the membrane; e.g., see Figure 6c. The heterogeneity in the deposits that adhered to the membrane surface after the cleaning cycle was reinforced by the SEM-EDS analysis shown in Figure 7. Nonetheless, it seems that the impact of these deposits on the distillate flux obtained after the cleaning cycle is negligible compared to the case of distillation in a brand-new membrane; e.g., see Figure 3 and Figure 4. This result suggests that the deposits that adhered to the membrane surface after the cleaning process occupied a relatively small area. As future work, we suggest investigating if salt-crystal removal in the membrane pores can be further studied with SEM, as well as using it to thoroughly map cross sections of the membranes.

## 5. Conclusions

This work presents a simple mathematical model that aims to predict distillated water fluxes in DCMD when inorganic fouling (scaling) occurs. This model uses a heat and mass transfer formulation for prediction of the distillate flux under steady-state conditions, and it is combined with the cake-filtration theory to represent the distillate fluxes after the onset of membrane fouling. Distillate fluxes of ~12–14 kg m^−2^ h^−1^ were obtained with brand-new or cleaned membranes, whereas after the onset of membrane fouling, these fluxes rapidly decreased to zero. Additionally, recovery rates ranged between 25–26% (Experiment 2) and 34–42% (Experiment 1), and the electrical conductivity of the distillate channel remained at values lower than 1 µS cm^−1^ during all the experiments. From the experimental observations and the modeling, exercise we found that: (i) the critical time (*t_c_*) obtained for each subset of experiments was relatively constant; (ii) the precipitation reaction constant ($k_{pr}$) is conditioned by the physicochemical interaction between the solution and the membrane; and (iii) the $k_{f}$ coefficient, which represents the rate of flux decline after membrane fouling, depends on the flow conditions and the precipitated compound. Additionally, SEM was found to be an important tool to qualitatively assess the conditions of the membranes.

As the model results agree well with observed data, the cake-filtration theory can be used to represent water flux decline in membranes prone to inorganic fouling. However, the proposed model has limitations that must be addressed in future investigations to validate it under a wider range of operating conditions, with different feed solutions, and for membranes composed of other materials. For instance, organic, biological and/or colloidal fouling are phenomena that deserve attention, as they typically occur under real conditions. Additionally, an assessment of the impact of the cleaning agents on membrane properties must be explored in the future. For this, SEM images should be explored to investigate if salt crystal removal in the membrane pores can be observed, as well as to map the cross sections of the membrane to study how fouling occurs.

The practical aspect of the proposed model is that it can provide valuable information to be used in scaling up the MD system, as well as for their operation. Predictive models such as that presented in this work can help to check and replace membranes at optimal times and improve the overall efficiency of this water-treatment system by minimizing interruption times associated with cleaning.

## Figures and Tables

**Table 1:** Description of the experiments reported in this investigation.

Parameters	Experiment 1	Experiment 2
Feed solution composition	20.5 g CaCl_2_∙2H_2_O 18.7 g MgSO_4_∙6H_2_O 4.5 g NaCl	20.5 g CaCl_2_∙2H_2_O 10.8 g Na_2_∙SO 1.2 g NaCl
Feed solution volume (L)	4	4
Feed salinity (%)	1.09	0.81
Cleaning solution composition	20 g EDTA 5 g NaOH	11.7 g NaCl
Cleaning solution volume (L)	2	2
Time of cleaning (min)	30	30
Rinse volume (L)	12	12
Number of cleaning cycles	2	5
Pa (kPa)	94	94
P (kPa)	101.3	101.3
Feed channel temperature (°C)	38.8	40.0
Distillate channel temperature (°C)	20.8	20.0
$h_{f}$ (W m^−2^ °C^−1^)	1.20 × 10^4^	1.21 × 10^4^
$h_{d}$ (W m^−2^ °C^−1^)	9.55 × 10^3^	9.46 × 10^3^
K (m s^−1^)	1.89 × 10^−4^	1.90 × 10^−4^

**Table 2:** Results obtained in experiments 1 and 2.

Cleaning Cycle	Experiment 1				Experiment 2			
	$k_{pr}$ (kg h^−1^)	Error in precipitated salts (kg)	$t_{c}$ (h)	R (%)	$k_{pr}$ (kg h^−1^)	Error in precipitated salts (kg)	$t_{c}$ (h)	R (%)
New membrane	1.21 × 10^−3^	4.95 × 10^−10^	6.79	42	5.20 × 10^−4^	2.51 × 10^−10^	4.39	25
After first cleaning	9.34 × 10^−4^	1.45 × 10^−11^	6.31	37	6.81 × 10^−4^	1.64 × 10^−10^	5.00	25
After second cleaning	1.39 × 10^−3^	1.55 × 10^−10^	5.96	34	6.72 × 10^−4^	1.91 × 10^−10^	4.43	25
After third cleaning	-	-	-	-	1.13 × 10^−3^	2.37 × 10^−10^	4.09	25
After fourth cleaning	-	-	-	-	8.28 × 10^−4^	2.38 × 10^−10^	4.12	25
After fifth cleaning	-	-	-	-	1.12 × 10^−3^	1.81 × 10^−10^	4.73	26

## Data Availability

The data used in this research can be requested to the corresponding author.

## Supplementary Materials

The following supporting information can be downloaded at: https://www.mdpi.com/article/10.3390/membranes12020157/s1.

## Appendix A. Estimation of the Heat and Mass Transfer Coefficients Used in the Model

For laminar flow, Equations (A1)–(A5) were used to determine the heat and mass transfer coefficients for each channel in the membrane module [27,57]:

(A1) $$h_{f}=Nu_{f}\frac{k_{f}}{d_{h}}=\left[1.86{\left({Re}_{f}{Pr}_{f}\frac{d_{h}}{L}\right)}^{1/3}\right]\frac{k_{f}}{d_{h}},$$

(A2) $$h_{d}=Nu_{d}\frac{k_{d}}{d_{h}}=\left[1.86{\left({Re}_{d}{Pr}_{d}\frac{d_{h}}{L}\right)}^{1/3}\right]\frac{k_{d}}{d_{h}},$$

(A3) $$K=Sh_{f}\frac{D_{f}}{d_{h}}=\left[1.86{\left({Re}_{f}{Pr}_{f}\frac{d_{h}}{L}\right)}^{1/3}\right]\frac{D_{f}}{d_{h}},$$

(A4) $$Pr=\frac{\nu }{\alpha }$$

(A5) $$Re=\frac{u_{f}d_{h}}{\nu }$$

where Nu (-), Sh (-), Pr (-), and Re (-) are the Nusselt, Sherwood, Prandtl, and Reynold numbers, respectively. Subscripts f and d correspond to the feed and distillate sides, respectively; $d_{h}$ and L (m) are the hydraulic diameter and the length of the channels in the membrane module, respectively; $D_{f}$ (m^2^ s^−1^) is the diffusion coefficient of the solute; k (W m^−1^ °C^−1^) is the thermal conductivity of the liquid streams; ν (m^2^ s^−1^) is the fluid’s kinematic viscosity; α (m^2^ s^−1^) is the fluid’s thermal diffusivity; and u (m s^−1^) is the average velocity in the channels of the membrane module.

For turbulent flow, the heat and mass transfer coefficients were estimated based on Equations (A6)–(A9) [27]:

(A6) $$h_{f}=Nu_{f}\frac{k_{f}}{d_{h}}=0.023\left(1+\frac{6D_{h}}{L}\right)Re_{f}^{0.8}Pr_{f}^{1/3}\frac{k_{f}}{d_{h}},$$

(A7) $$h_{d}=Nu_{d}\frac{k_{d}}{d_{h}}=0.023\left(1+\frac{6D_{h}}{L}\right)Re_{d}^{0.8}Pr_{d}^{1/3}\frac{k_{d}}{d_{h}},$$

(A8) $$K=Sh_{f}\frac{D_{f}}{d_{h}}=0.023\left(1+\frac{6D_{h}}{L}\right)Re_{f}^{0.8}Sc_{f}^{1/3}\frac{D_{f}}{d_{h}},$$

(A9) $$Re=\frac{u_{d}d_{h}}{\nu }$$

where Sc (-) is the Schmidt number. The heat transfer correlations presented above were chosen as they resulted in the lowest discrepancies (approximately 9%) between experimental and calculated overall heat transfer coefficients [27,57].
